# P137: Effectiveness of a hand hygiene improvement program in doctors: active monitoring and real-time feedback

**DOI:** 10.1186/2047-2994-2-S1-P137

**Published:** 2013-06-20

**Authors:** SR Kim, MH Cho, WJ Kim, JY Song, HJ Cheong

**Affiliations:** 1 Infection Control Unit, Korea University Guro Hospital, Seoul, Korea, Republic Of; 2 Division of Infectious Diseases, Department of Internal Medicine, Korea University Medical College, Seoul, Korea, Republic Of

## Introduction

Hand hygiene is the single most important intervention to combat infections in diverse health care settings. However, adherence to hand hygiene practice remains low among health care workers, especially in doctors.

## Objectives

The aim of study was to promote hand hygiene compliance in doctors.

## Methods

Hand hygiene practice was monitored by trained observers every three months. We provided performance feedback to doctors using short message service and posters. Also, we conducted the self-check questionnaire survey of hand hygiene performance quarterly. We asked doctors whether they clean hands before contact with patients and clean/aseptic procedures in wards and out-patient room.

## Results

The overall hand hygiene compliance rate increased from a baseline of 49.7% in fourth quarter of 2011 to 82.3% in fourth quarter of 2012 (*p*<.001). Response rate of self-check questionnaire increased from a baseline of 82.9% to 93.8% (*p*=.004). Compliance with hand hygiene was higher in Wards and higher before clean/aseptic procedures than before contact with patients.

## Conclusion

The self-check questionnaire survey of hand hygiene performance would be useful to increase awareness of hand hygiene, and performance feedback improved the compliance rates in a sustained manner.

## Disclosure of interest

None declared

